# Gap Junctions and Epileptic Seizures – Two Sides of the Same Coin?

**DOI:** 10.1371/journal.pone.0020572

**Published:** 2011-05-31

**Authors:** Vladislav Volman, Matjaž Perc, Maxim Bazhenov

**Affiliations:** 1 Computational Neurobiology Laboratory, The Salk Institute for Biological Studies, La Jolla, California, United States of America; 2 Center for Theoretical Biological Physics, University of California San Diego, La Jolla, California, United States of America; 3 Department of Physics, Faculty of Natural Sciences and Mathematics, University of Maribor, Maribor, Slovenia; 4 Department of Cell Biology and Neuroscience, University of California Riverside, Riverside, California, United States of America; Indiana University, United States of America

## Abstract

Electrical synapses (gap junctions) play a pivotal role in the synchronization of neuronal ensembles which also makes them likely agonists of pathological brain activity. Although large body of experimental data and theoretical considerations indicate that coupling neurons by electrical synapses promotes synchronous activity (and thus is potentially epileptogenic), some recent evidence questions the hypothesis of gap junctions being among purely epileptogenic factors. In particular, an expression of inter-neuronal gap junctions is often found to be higher after the experimentally induced seizures than before. Here we used a computational modeling approach to address the role of neuronal gap junctions in shaping the stability of a network to perturbations that are often associated with the onset of epileptic seizures. We show that under some circumstances, the addition of gap junctions can increase the dynamical stability of a network and thus suppress the collective electrical activity associated with seizures. This implies that the experimentally observed post-seizure additions of gap junctions could serve to prevent further escalations, suggesting furthermore that they are a consequence of an adaptive response of the neuronal network to the pathological activity. However, if the seizures are strong and persistent, our model predicts the existence of a critical tipping point after which additional gap junctions no longer suppress but strongly facilitate the escalation of epileptic seizures. Our results thus reveal a complex role of electrical coupling in relation to epileptiform events. Which dynamic scenario (seizure suppression or seizure escalation) is ultimately adopted by the network depends critically on the strength and duration of seizures, in turn emphasizing the importance of temporal and causal aspects when linking gap junctions with epilepsy.

## Introduction

Most of the communication between the neurons in the brain of an adult animal is achieved by means of chemical synapses [1]. In the cortex for example, a typical neuron can get as many as 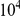 synaptic connections from, and communicate its spike to, other neurons, thus making synaptic transmission a ubiquitous mode of information transfer. Consequently, over the years many successful “synaptic theories” were developed to explain a variety of phenomena, such as the working memory [2], long-term plasticity and memory formation [3], but also pathological conditions such as the schizophrenia [4] and the epilepsies [5]. In particular, one of the widely accepted theories attributes epileptic seizures to the shift of synaptic balance toward excitation in conditions of impaled inhibition or augmented excitation [5]–[7].

Besides chemical synapses, neurons can also form direct electrotonic connections with their peers via electrical synapses, or so called gap junctions [8], [9]. Conceptually, a network of neurons coupled by gap junctions has often been likened to excitable reaction-diffusion (RD) media [10]. In RD systems, a substance (usually chemical) spreads by diffusion from one excitable site to its neighbors where it can be regenerated by a reaction, and the process repeats itself, resulting in the propagation of regenerative waves through the medium [11], [12]. The speed of wave propagation is mostly limited by the characteristic reaction time. In RD neuronal networks, membrane voltage plays the role of a “diffusible chemical” and gap junctions play a role of “diffusion” providing coupling of membrane voltages between neighbor neurons; since the spike width (“reaction time”) is very short (∼ 1 ms) the “voltage wave” can quickly engage neurons that are relatively far apart in space. Indeed, computational modeling studies supported by mathematical analysis suggested that a network of neurons coupled by gap junctions can support collective activity in the form of waves that are generated spontaneously and propagate through the network [13]. The existence of such dynamical state requires the coupling by gap junctions to be sparse and strong [13]. Gap junctions also play an important role in promoting synchronization in networks of inhibitory interneurons, which is believed to be necessary for the generation of collective oscillatory activity in the gamma band (30–80 Hz) [14], [15].

The role of gap junctions in synchronization of neuronal ensembles and propagation of neuronal activity led to suggest that this mode of communication could be a critical factor in the emergence of some forms of pathological activity, such as the epileptic seizures, as reviewed by Perez-Velazquez and Carlen [16]. The emergence of epileptic activity in the brain usually reflects an imbalance between depolarizing influences (which tend to excite the neuron and result in more intense spiking) and hyperpolarizing influences (which suppress spiking activity by keeping the membrane potential sufficiently below the threshold for spike generation). Any change in the excitation-inhibition balance that favors excitation can in principle promote seizure-like activity [17], [18]. Because gap junctions enhance synchronization in networks of neurons, this can potentially lead to the elevation of excitatory activity and thus promote epileptogenesis. The hypothesis about the putative role of neuronal gap junctions in seizures is further supported by the observation that the expression of gap junctions is enhanced in epileptic slices.

However alluring is the hypothesis linking neuronal gap junctions to epileptic seizures, there remain several caveats. Firstly, in experimental models the expression of gap junctions is significantly increased *following* the epileptic-like intense stimulation of otherwise “normal” slice [19], [20]. This often overlooked fact puts a question mark on the causal role of direct electrical coupling in seizure onset. If addition of gap junctions always promotes seizures, then why does the network respond to the imposed seizure-like stimulation by adding more gap junctions? Secondly, the expression of neuronal gap junctions is highest in early development, progressively decreases as synaptic connectivity patterns are established, but is not reduced to zero even in mature and “healthy” networks [9], [21], as would be expected if gap junctions played a purely pathological role in shaping network dynamics. This suggests that, at least in some cases, an abundance of gap junctions could represent an adaptive response of a network to aberrant patterns of overly synchronized and intense activity (as occurs in immature networks) rather than being an anatomical aberration in itself. Finally, computational models of reaction-diffusion systems usually assume sparse, strong, and topologically regular connectivity which promotes synchronization and wave propagation. However, in real gap-junction coupled neuronal networks, both the number of neurons to which a given neuron connects via electrical synapses, and the strength of this connection (unitary conductance) can vary greatly across the network [8], [22]. Hence, one must be careful in carrying the results derived for “classical” RD systems over to gap junction coupled neuronal networks with heterogeneous patterns of electrical connectivity.

Here, we have devised a minimal biophysically plausible computational model of neuronal network in order to investigate the role of neuronal gap junctions in maintaining stability in network dynamics. We show that for physiological levels of spiking activity that are typical for a “healthy” cortex (∼5 Hz for pyramidal neurons and ∼10 Hz for interneurons), gap junctions can serve as a mechanism that would stabilize neuronal network dynamics in response to relatively mild perturbations in neural activity. This stabilizing effect relies critically on the topological connectivity, strength of individual gap junction connection, and strength of the perturbation to neural activity; with strong perturbations, gap junctions promote collective high-frequency oscillations indicative of epileptic activity. By topological connectivity we mean here the topology of the “connectivity space”, which defines the properties of signal (or perturbation) propagation through the network of connected neurons. Based on these observations, we propose a solution to the apparent paradox regarding the enhanced expression of gap junctions that follows seizure-like stimulation [19], [20]. Using model simulations, we show that initial post-seizure addition of gap junctions can suppress the pathological seizure-like excitation of the neuronal ensemble. This suggests that an experimentally observed increased post-seizure expression of gap junction channels could represent an adaptive response of the neuronal network to the potentially pathological perturbation of activity. If the activity perturbation is relatively weak and transient, such adaptive responses can suppress seizure initiation. Conversely, if the perturbation is strong and persistent, an initially activity-suppressing effect of enhanced gap junctional connectivity can revert and lead to seizure escalation (with gap junctions engaging neurons in synchronous firing). Our results thus imply that gap junctions could be either deleterious or beneficial, depending on the strength and the duration of the applied perturbation. Thus, we suggest that the hypothesis linking gap junctions to epileptic seizures should be revised to account for temporal and causal aspects.

## Results

### Topological connectivity determines the impact of unitary conductance on noise-driven activity

**Figure 1:**
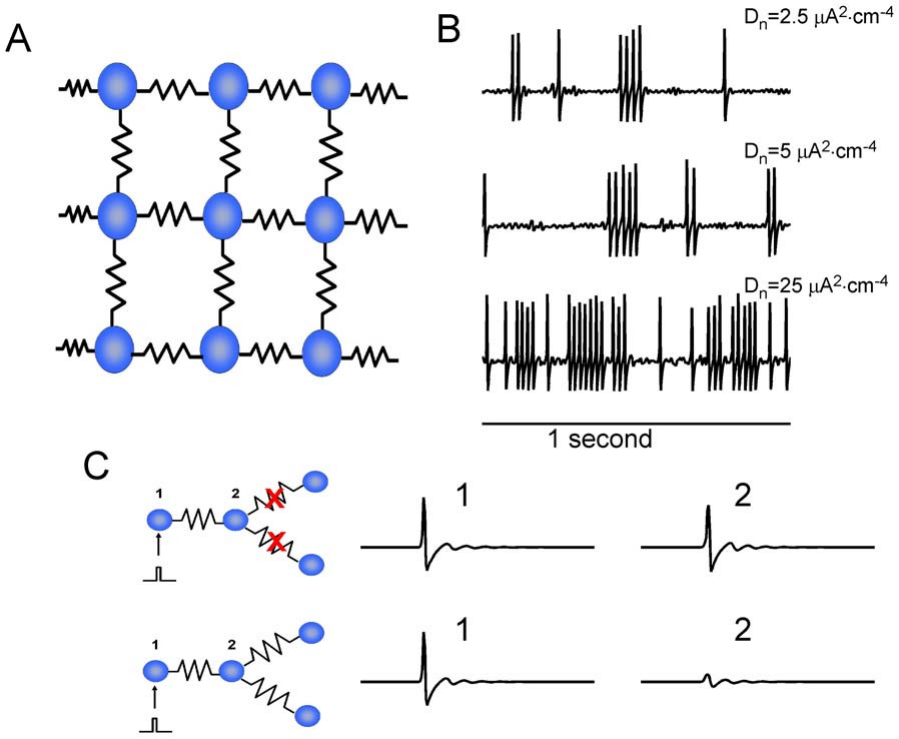
Characteristics of gap-junction coupled neuronal network model. **A** Schematic presentation of network connectivity, for Z = 4 (four gap junction connections per model neuron). **B** Intensity of stimulation current determines the rate of neuronal spiking. Top panel: 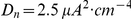. Middle panel: 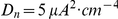. Bottom panel: 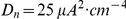. These traces of membrane potential are for an isolated model neuron (not connected to other model neurons). **C** Strong topological coupling by gap junctions can impede signal transmission. Model neuron 1 was stimulated by a brief step-like constant current which was sufficient to generate a spike. Depending on the extent of its gap junction connectivity, model neuron 2 could either generate a spike or responded with a sub-threshold voltage to the spike event in model neuron 1. The intensity of stimulation current was set to zero in this example. The strength of functional coupling was 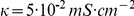 (10 fold higher as compared to the baseline network model, to compensate for the lack of noisy stimulation current).

We considered 2D networks of 50×50 model neurons coupled with gap junctions, with periodic boundary conditions (Figure 1). Neuronal dynamics were described using the well-studied Morris-Lecar model [23] that was slightly modified by Prescott et al. [24] to account for correct biological mechanisms of action potential generation. This simplified model provides an optimal balance between realistic electrical properties of neuron and computational performance that allows simulations of large-scale 2D networks. In the baseline model, each model neuron was coupled by gap junctions to its Z nearest neighbors (Z ranged from 4 to 24, as described in Methods); pattern of gap junction connectivity determined properties of spike propagation through the network (Figure 1C). Random (see Methods for details) external input with amplitude determined by parameter *D_n_* was applied to all the neurons to drive them beyond the spiking threshold. This external stimulation can be interpreted as a random synaptic input from the rest of the neuronal population that was not included in this network model.

**Figure 2:**
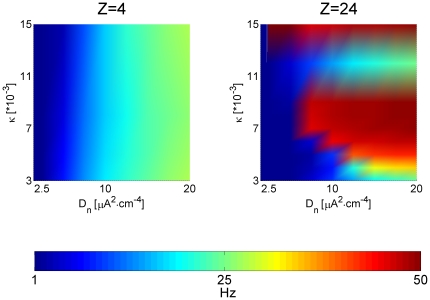
Topological connectivity determines the effect of driving noise and functional coupling on neuronal firing rate. Left: map of network-averaged firing rate vs. the strength of functional coupling (unitary conductance 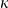, vertical axis) and the intensity of driving noise (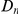, horizontal axis), for Z = 4. Right: map of network-averaged firing rate vs. the strength of functional coupling (unitary conductance 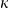, vertical axis) and the intensity of driving noise (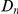, horizontal axis), for Z = 24. Color code is blue for low firing rates and red for high firing rates, and color scale is the same for both panels.

Just as synaptic connectivity is determined both by the number of connecting neurons (topological aspect) and the strength of synaptic weight between a particular pair of neurons (functional aspect), the electrical connectivity is determined by the number of neurons that establish gap junctions with a particular neuron (topological aspect, parameterized by the number of neighbors Z in our model), and the unitary gap junction conductance and the number of gap junctions between a given pair of neurons (functional aspect, parameterized by the strength of coupling 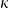 in our model). How do the topological and the functional aspects of gap junction connectivity determine the patterns of neuronal activity? Figure 2 shows the dependence of network-averaged firing rate on the strength of coupling (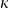) and on the intensity of stimulation current (*D_n_*). For low topological connectivity (Z = 4, left plot) the strength of coupling only weakly affected the dependence of the firing rate on the stimulation intensity. On the other hand, in highly interconnected network (Z = 24, right plot) the “firing rate-driving noise” relation was critically shaped by the value of parameter 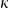. The critical intensity of stimulation current for which the transition from low to high rate firing occurred generally moved to the left for higher 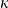 (note, however, a “gap” for an intermediate coupling strength 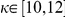, indicating a transition from the regime of stimulus-driven activity to the regime in which spiking actively spreads through the network via strong gap junctions). Thus, for a given strength of functional coupling 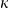 (gap junction conductance) the topological coupling (number of neighbors) could set the context for the intensity of noisy synaptic-like stimulation that was needed in order to drive the network to relatively high firing rates (>20 Hz). In particular, in the regime of weak coupling (small 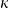), stronger topological connectivity (high Z) tended to suppress stimulus-driven activity over a wide range of stimulation intensities (compare, e.g., left and right plots in Figure 2 for 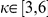). The implication of this regime (very weak expression of gap junctions, as appears in “healthy” mature cortex) to the regulation of collective activity is discussed below.

### Strong topological connectivity inhibits collective activity in neuronal network

**Figure 3:**
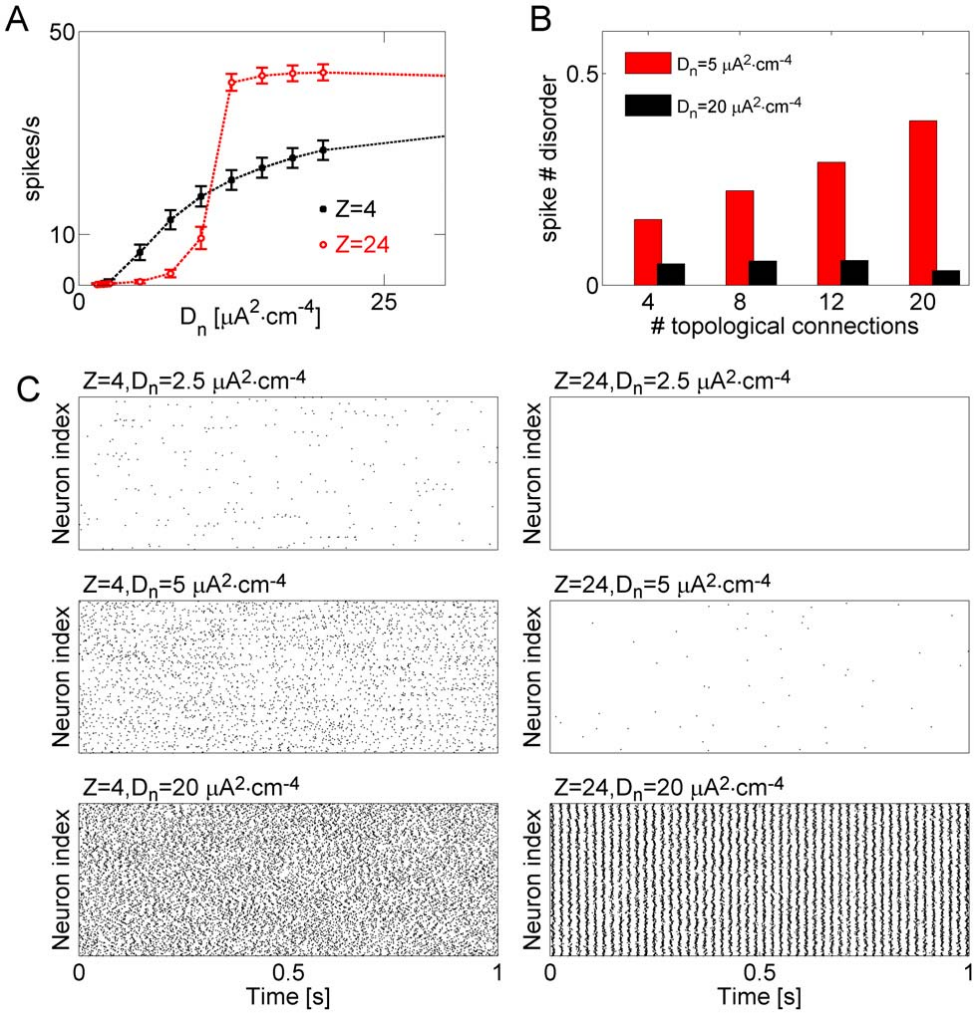
Strong topological connectivity can suppress spiking activity in a network of neurons coupled by gap junctions. **A** Network-averaged firing rate (mean
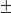
S.E.M.) vs. the intensity of driving noise, for different scenarios of topological connectivity: Z = 4 connections per neuron (black closed squares); Z = 24 connections per neuron (red open circles). The firing rate was computed over the time window of 20 seconds. The coupling strength was 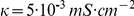. **B** Spike number disorder vs. the number of topological connections, for different driving noise intensities: 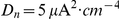 (red); 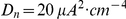 (black). **C** Sample raster plots of network activity, for different scenarios of topological connectivity and driving noise intensities: 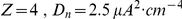 (left top); 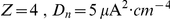 (left middle); 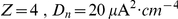 (left bottom); 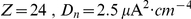 (right top); 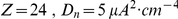 (right middle); 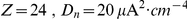 (right bottom).

Figure 3A shows the dependence of mean and standard deviation of averaged (over network) neuronal firing rate on the intensity of the driving current, 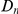, for different patterns of topological connectivity. While for sufficiently intense inputs, intense gap junction connectivity (more pair-wise connections between any 2 model neurons) led to more intense and more regular firing (Figure 3A), this effect was opposite for the low driving current (described by lower 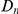). Effect of the connectivity pattern (Z) on the firing rate inverted at 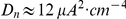. Thus, for weak driving current the higher number of topological connections between the neurons appeared to suppress the stimulus-driven spiking (Figure 3A). As was explained in [25] this suppressing effect is attributed to the faster relaxation of sub-threshold perturbations in a presence of gap junction coupling between neurons that allows depolarizing currents to “escape” from perturbed neurons to its neighbors. Indeed, for relatively weak electrical coupling strength and large number of neighbor neurons, the effective leak conductance of a neuron is increased; the rheobase (a minimal constant current that is needed to evoke spiking) is increased as well. Under these conditions, a perturbation to a given neuron is effectively shared by all neighbor neurons and the effect of the perturbation is diluted, reducing the chances to generate action potential. This mechanism promotes the stability against relatively modest perturbations (below the rheobase which is determined by the local gap junction connectivity). In contrast, for supra-rheobase perturbations, the presence of gap junctions aids in activity propagation and network synchronization.

The stabilizing effect of the strong topological coupling in the regime of weak driving current had additional implications for collective neuronal activity; faster decay of sub-threshold perturbations was expected to result in more failures in signal propagation thus increasing the spike number disorder measure (see Methods). We verified this hypothesis by computing the (binned) intensity of collective activity for different patterns of connectivity and stimulation current intensity (Figure 3B). For weak stimulation (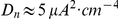), stronger coupling between the model neurons significantly increased the spike number disorder measure (Figure 3B). In contrast, the spike number disorder measure remained nearly constant as a function of topological coupling when the network was driven by high intensity current (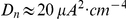) (Figure 3B). Further visual inspection (Figure 3C) shows that in the weak topological connectivity regime (Z = 4, left panel in Figure 3C) increasing the intensity of stimulation gradually increased the firing rate of model neurons while the collective activity remained largely asynchronous. By contrast, in highly interconnected network (Z = 24, right panel in Figure 3C) applying the same driving current intensity first resulted in only minimal changes to the network firing rate but as the input intensity increased led to high-frequency synchronous bursts qualitatively similar to inter-ictal epileptiform discharges.

These results demonstrate that under certain circumstances (weak functional coupling, manifested physiologically as small unitary conductance) strong topological connectivity by gap junctions can help down-regulate collective neuronal activity. Stated in other words, in networks coupled by electrical synapses with weak permeability of individual gap junctions, local structure of topological connectivity can be a network stabilizing factor, an implication which could have far reaching global effects when long-range chemical synaptic interaction is explicitly added. Chemical synapses provide a way for fast, long-distance neuronal communication; thus, local perturbations of electrical activity can quickly spread and ignite the network. In the situation like this, the local characteristics of gap junction connectivity can play a pivotal role in defining the local response to perturbations. In what follows, we investigated how local variations in topological connectivity of electrical coupling and stimulation intensity (that may be interpreted as the strength of perturbations due to long-range chemical synaptic communication) change local and global dynamics in a network of model neurons coupled by gap junctions.

### Hot-spots of intense activity are associated with weakly coupled neurons

Epileptic activity often arises following traumatic brain injury (TBI) which can impair synaptic connectivity and thus affect the local excitation/inhibition balance [26], [27]. Given this causal role of synaptic connectivity changes in the onset of seizures, we wanted to see whether the trauma-like changes in gap junction connectivity would have any similar effect. To this end we considered a simple “trauma scenario” in which the gap junction connectivity in a predefined subnetwork (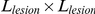 block of neurons) of highly interconnected network (Z = 24) was reduced compared to the connectivity in the rest of the network. The reduction in the connectivity inside the lesioned subnetwork was parameterized by 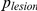 which corresponded to the probability to destroy an existing connection between a pair of model neurons inside the lesioned block. Thus, for 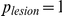 all the neurons inside the square 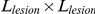 block (total 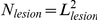 neurons) were completely disconnected from each other, but retain their gap junction connections with the “intact” neurons in the rest of the network. Since we were interested in the effect that the structural change in gap junction connectivity can have on the emerging activity, the intensity of the stimulation current was kept constant throughout these simulations (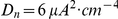).

**Figure 4:**
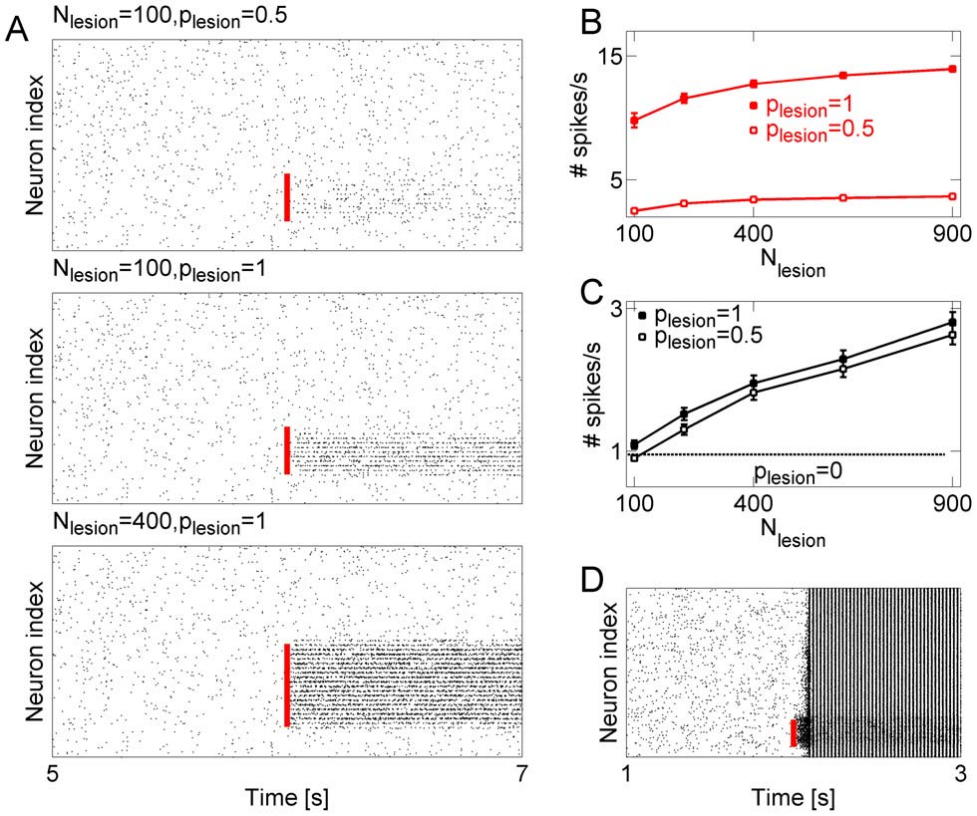
Hot-spots of activity are associated with weak topological connectivity. **A** Raster plots showing the response of model neuronal network to the localized lesion of gap junction connectivity. The extent of lesion is parameterized by the probability 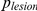 to destroy a connection between a pair of model neurons in the predefined area 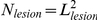. The timing of lesion is marked with red line. Top panel: 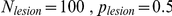. Middle panel: 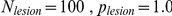. Bottom panel: 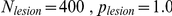. Other parameters are: 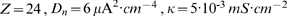. **B** Averaged firing rate (mean
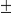
S.E.M.) in the lesioned subnetwork vs. the size of the lesioned subnetwork. Closed squares: 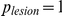. Open squares: 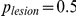. In all cases 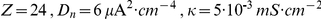. Firing rate was computed over the time window of 20 seconds. **C** Averaged firing rate (mean
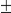
S.E.M.) in the intact subnetwork, vs. the size of the lesioned subnetwork. Closed squares: 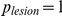. Open squares: 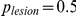. In all cases 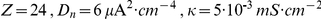. For comparison, dashed line is the averaged neuronal firing rate in baseline conditions (model network with fully intact connectivity). Firing rate was computed over the time window of 20 seconds. **D** Raster plot showing the response of a model network with electrical and chemical synapses to the localized breach in gap junction connectivity. Disruption to gap junction connectivity was applied at time T = 2 seconds and is marked with red line. Chemical synaptic connections were added to the model as described in Methods.

As Figure 4 shows, lesioning of gap junction connectivity at time T = 6 seconds led to increase in spiking activity for model neurons in the traumatized subnetwork. In Figure 4A, the time at which the connectivity was lesioned and the extent of lesioned region are marked with red line. Firing rate of model neurons in the traumatized subnetwork was higher for larger lesions (higher values of 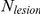 in Figure 4B) and increased for stronger disruption of gap junction connectivity inside the traumatized region (higher value of p_lesion_ in Figure 4B). Interestingly, the firing rate of the neurons with intact connectivity also increased (Figure 4C), which was largely attributed to increase of activity of neurons near the border with traumatized region, but propagation of high frequency activity through the intact network was not supported (Figure 4A bottom panel). Overall, following the lesion, the firing rate of lesioned neurons could increase up to 10-fold as compared with the activity in the baseline model before lesion (Figure 4B,C). Thus, localized lesions of gap junction connectivity can create localized hot-spots of intense activity.

To test if the model prediction is correct in the network with mixed synaptic and electrical connectivity, we extended our model by including synaptic interactions (see Methods for description of this simulation). Similar to the previous simulations, the intact network displayed stable asynchronous activity. Following the local lesion of gap junction connectivity, firing rates were increased, activities of individual neurons became synchronized and were carried to other (“healthy”) parts of the network, resembling seizure-like dynamics (Figure 4D).

### Adaptive increase in gap junction number can mitigate the response to mild perturbations

We showed earlier that the localized lesion of gap junction connectivity could result in a localized strong (up to 10-fold) increase in the firing rate of affected neurons. Chemical synaptic signaling could in principle communicate this increased firing rate to the other parts of the network and thus create a perturbation of neuronal excitation there. Such increase of “external” drive to a part of the network can be simulated in our model by changing intensity 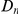 of stimulation current. The last can therefore be interpreted as increase in random synaptic drive from the parts of the network that are not explicitly modeled here. Below we will ask if a localized change in gap junction content may help to mitigate the potentially pathological effect of such random input increase.

**Figure 5:**
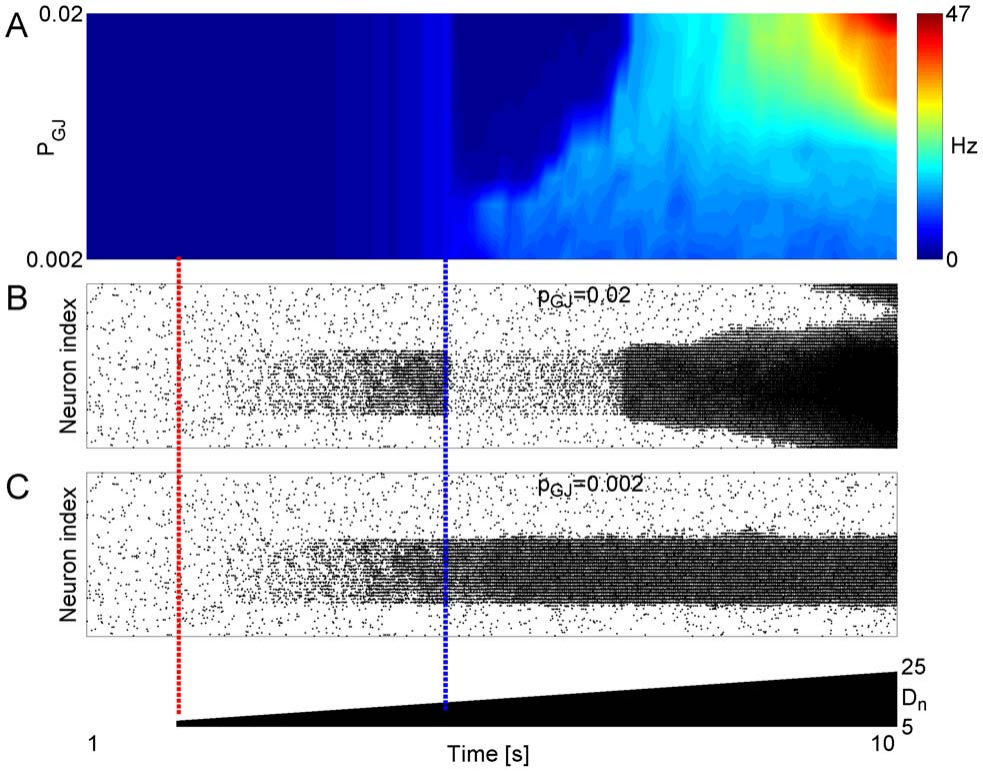
Increased gap junction connectivity can mitigate the response to mild perturbation. **A** Color panel is the surface plot of firing rate (averaged over non-overlapping bins of 100 ms and over all model neurons) vs. the probability 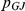 to establish a new gap junction between a pair of previously unconnected neurons from the affected area (
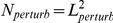
,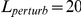). Color code is blue for low firing rate and red for high firing rate. Horizontal axis is simulation time ([0–10] seconds). Background noise intensity 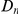 for the set of 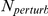 model neurons was perturbed at 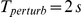 (dashed red line through Panels B,C), and progressively increased to achieve 5-fold higher values at time 10 seconds (scale bar in lowest panel). Gap junction connectivity for 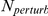 model neurons was increased (as specified by 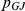) at time 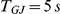 (dashed blue line through Panels B,C). **B** Raster plot of network's activity for 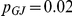. Other parameters: 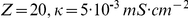. **C** Third panel: Raster plot of network's activity for 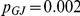. Other parameters: 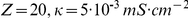.

To address these questions, we considered a simple scenario in which model neurons in the predefined sub-network (square 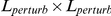 block, total number of perturbed neurons 
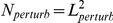
) were subjected to higher intensities of stimulation current as compared to the baseline input intensity applied to the neurons in the rest of the network. The change of the stimulus intensity was initiated at time 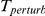 (marked with red dashed line in Figure 5), and the intensity of the stimulation was gradually increased (Figure 5, bottom panel). Overall, starting from the time of the initial perturbation, the background current intensity increased 5-fold compared to its baseline value (from 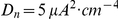 to 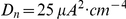). To address the possible regulatory effect of gap junction connectivity, at time 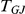 (marked with blue dashed line in Figure 5, 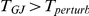) new gap junction connections were formed between the model neurons in the perturbed sub-network, such that the probability to create a new gap junction between a pair of previously unconnected neurons was 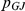.

In Figure 5A, the firing rate (average over non-overlapping bins of 100 ms and over all neurons in the network) is shown for different values of 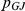 and different stimulation current intensities. As expected, higher stimulus intensity always led to more intense neuronal firing. For relatively mild perturbation, its effect on the firing rate could be offset by adding more gap junctions between previously unconnected model neurons in the perturbed sub-network (Figure 5B, middle section). However, if the perturbation of neuronal excitation was further increased, a more interconnected sub-network could become a focus of intense activity that spread through the entire network (Figure 5B, rightmost section). When fewer new gap junction connections were added, it had almost no stabilizing effect of the network firing rate (Figure 5C). Thus, sufficiently strong increase in gap junction connectivity in response to the increased levels of activity (as occurs in experimental models of evoked seizures) has two facets – it can either bring the activity back to the normal regime or contribute to seizure initiation. Which one of the routes is ultimately taken depends on the strength, pattern and duration of the perturbation applied to the network.

**Figure 6:**
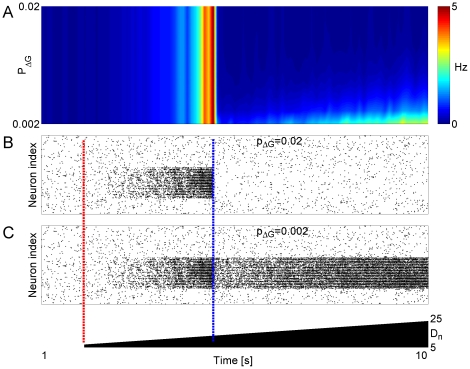
Changes in network topology and membrane conductance underlie the overall effect of gap junction connectivity on the network stability. **A** Color panel is the surface plot of firing rate (averaged over non-overlapping bins of 100 ms and over all model neurons) vs. the probability 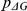 to increase the leak conductance by 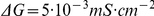 in each one of the model neurons from the affected area (
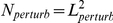
,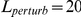) that did not previously share gap junction connection. Color code is blue for low firing rate and red for high firing rate. Horizontal axis is simulation time ([0–10] seconds). Background noise intensity 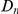 for the set of 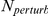 model neurons was perturbed at 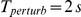 (dashed red line through Panels B,C), and progressively increased to achieve 5-fold higher values at time 10 seconds (scale bar in lowest panel). Membrane leak conductance for 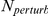 model neurons was increased (as specified by 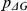) at time 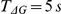 (dashed blue line through Panels B,C). **B** Raster plot of network's activity for 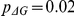. Other parameters: 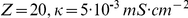. **C** Third panel: Raster plot of network's activity for 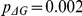. Other parameters: 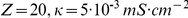.

Because the basic mechanism by which gap junctions affect network stability is a change in the value of rheobase current (and thus a change in the excitability of individual neurons), the results presented in Figure 5 could be a consequence of reduced excitability of model neurons after adding new gap junctions. Alternatively, both altered neuronal excitability and change of the efficiency with which a perturbation of activity can propagate through the network with different density of connections might be involved. To test this question we performed the following test. Same network (as the one studied in Figure 5) was considered, and the same perturbation (time of perturbation marked with red dashed line in Figure 6), was applied to the network as in Figure 5. At time 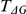 (marked with blue dashed line in Figure 6, 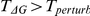), pairs of model neurons in perturbed sub-network were picked at random with probability 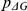, and membrane conductance of each one of these model neurons was increased by 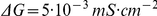 (which corresponds to the conductance of individual gap junction in our model). In this way, we obtained a network in which pattern of gap junction connectivity was the same one as in the baseline “healthy” network, but membrane conductances of neurons in perturbed sub-network were increased by the same amount as would have occurred if actual new gap junctions were added. This allowed us to separate the effects of increased membrane conductance from the effects of increased connectivity.

A brief glance at Figure 6 discloses the dramatic effect that the gap junction connectivity (rather than a mere increase in membrane conductance) has on network dynamics. When leak conductance of neurons was increased by the same amount as would have occurred if actual gap junctions were added to the network, the firing rate was stabilized for a wide range of perturbation intensities. Scenarios that led to epileptic like outbreak of activity in the model with actual gap junctions present now resulted in stable dynamics (compare firing rates in upper right corner of top panel in Figures 5,6). Thus, indeed, stabilizing effect of gap junctions can be attributed to the increase in membrane conductance (decrease in input resistance) making neurons less excitable. In space of the model parameters, gap junctions stabilize network dynamics for low connectivity patterns (low 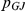) or weak external perturbations (low *D_n_*). In the limit of high 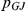 and high *D_n_* (top right corner in the Fig. 5A), however, gap junctions breached dynamical stability by means of higher connectivity.

## Discussion

As was first shown by Kepler et al. [28], resistive coupling can significantly affect the frequency of a neural oscillator in a way that depends on several parameters, such as the strength of coupling (conductance), the state of a neuron to which the given neural oscillator is coupled, and the sub-threshold dynamics of the oscillator. Our observations extend the conclusions of Kepler et al. [28] to networks of noise-driven model neurons with realistic firing properties, and highlight another aspect critical for collective activity – the topological connectivity. We identified a special regime in which strong topological connectivity can dramatically suppress the collective noise-driven activity. This occurred because topologically strong (large number of contacts) but functionally weak (relatively weak individual contacts) electrical connections reduced input resistance of the model neurons and, therefore, enforced fast relaxation of sub-threshold excitation, thus weakening the neuronal responsiveness to external stimulation [25]. However, the primary effect of increase in gap junction connectivity can be described as membrane conductance increase only under assumption that other neurons are far enough from spiking threshold. Since currents escape through gap junction to other neurons, not to the extracellular space, once many neurons are close enough to the spiking threshold, effect of gap junctions reverts and starts to mediate firing rate increase. Thus, in spatially extended networks of resistively coupled neurons driven by fluctuating current, the spatial profile of the current and the connectivity of the network (both its topological and functional aspects) can dramatically affect the emerging collective activity.

Our present study was restricted to investigations of dynamics in a network of neurons coupled only by gap junctions. Effects of chemical synaptic signaling were all lumped into the phenomenological “stimulation current”. This approximation allowed us to identify the contribution of gap junction coupling to the maintenance of dynamical stability in a network. In reality though, fast long-range signaling by chemical synapses can significantly affect the stability of the network dynamics, either by increasing the excitation (for example, by strong recurrent excitation) to the point of seizure outbreak, or by reducing the overall excitation through activation of the inhibitory interneurons. Existing models of epileptogenesis rely extensively on chemical synaptic interaction, and experimentally, epileptic seizures are eliminated by blocking chemical synaptic transmission. The sum effect of explicitly adding chemical synaptic transmission to our computational model would likely depend on the topology of synaptic connectivity and its relation to the local topology of gap junction connectivity; the relevant physiological data are not available at the moment. In the meantime, we note that the basic mechanism by which gap junctions can contribute to the maintenance of dynamical stability in the network does not depend on synaptic connectivity, therefore, our model predictions should still stand for the networks with mixed electrical and chemical connectivity.

**Figure 7:**
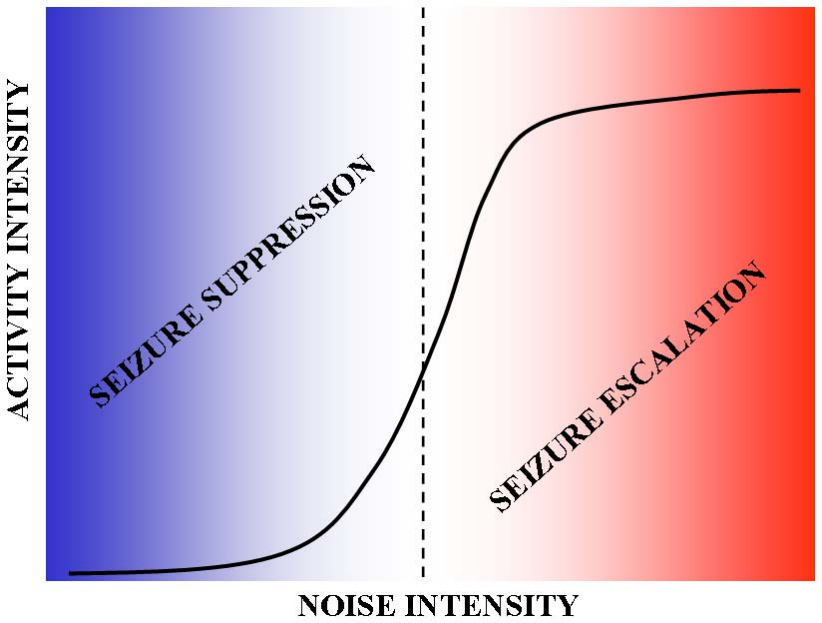
Schematic presentation of the effect that topological gap junction connectivity can have on the regulation of activity in networks that are prone to seizing. For mild perturbation of activity (low noise intensity, blue region), an increase in gap junction connectivity can offset the effect of perturbation and restore low firing rate, thus suppressing potential seizures. On the other hand, for strong perturbation (strong noise intensity, red region), an addition of gap junctions is likely to result in seizure escalation.

A significant body of data from in-vitro experiments and in-vivo measurements suggests that electrical coupling between neurons by means of gap junctions may play important role in information processing [29], [30], but also in the initiation and escalation of epileptic seizures [16]. Computational models suggest that gap junctions could be responsible for the generation of fast oscillations that precede seizures [31]. In physiological preparations, the expression of gap junctions was increased following the experimentally induced model of epilepsy [20]; thus, seizure in itself could be responsible for the increased gap junction content. Thus, while there is a consensus regarding the importance of gap junction communication in epilepsy, the causal link between enhanced gap junction expression and the emergence of seizures is still missing. Our studies suggest a possible new role for neuronal gap junctions that contrast a common view according to which this mode of communication is a pure epileptogenic factor promoting seizure activity. We posit that in experimental models of epileptic seizures, an initial increase in gap junction content between the neurons could represent an adaptive response by which the network tries to reduce the effects of transient aberrant neuronal activity. For relatively weak and transient perturbations of activity, such adaptive response can help to suppress the undesirable hyper-excitation; however, if the perturbation of activity is too strong and/or too persistent, an increase in gap junction content will lead to the escalation of seizure (Figure 7).

In the present study, we described neurons as one-compartmental entities, omitting the effects of dendritic integration altogether. While this assumption probably reflects fairly well the dynamics of electrotonically compact neurons, in other instances (such as for example is the case with pyramidal neurons in hippocampus) active dendritic conductances could profoundly affect the mechanisms of signal integration and spike generation. In fact, it was recently shown that individual dendritic branches of the same pyramidal neuron can operate quite autonomously with respect to the integration of synaptic signal; thus, pyramidal neurons are conceptually similar to the two-layer neural network, with dendritic layer processing synaptic input and somatic level evaluating the results of dendritic computation [32]. While these dendritic effects are not accounted for in our model, they would not change our qualitative conclusions regarding the role of topological connectivity; rather, the argument will become more “localized” pertaining now not to the entire neuron but to its individual dendritic branches. Exactly how the morphology of dendritic tree and the notion of “two-layer network” interact with topological considerations related to gap junction connectivity remains to be elucidated in further studies, using multi-compartmental modeling techniques.

As we argued in the present study, the topological connectivity of gap junction coupled neurons, as well as the intensity of background driving noise, can greatly affect the emerging network dynamics. While local gap junction connectivity was explicitly modeled in this study, much more spatially expanded synaptic inputs including both local and long-range connections from other network neurons as well as from neurons which were not explicitly modeled, was represented by background driving noise. With this assumption in mind, the above argument suggests that in the regime of weak gap junction coupling (as appears to be the situation in the “healthy” mature cortex) synaptic and gap junction connectivity can be delicately co-regulated to maintain physiologically normal activity. One point in support of this hypothesis is the observation that the expression of gap junctions is abundant in early development (when synaptic connections are not fully established) but is dramatically reduced as the network matures [21]. It is tempting to speculate that the relative expression of chemical vs. electrical synapses is subject to homeostatic co-regulation [33]; however, the mechanisms of this regulatory process at present remain unclear.

Although we focused here on the putative role of neuronal gap junctions in the generation of seizures, the conclusions of our present study are not limited to neuronal networks, but rather can be applied to any spatially extended excitable system with resistive coupling between its elements. Examples include networks of cardiac cells, liver cells, or glial cells of the brain. In particular, for networks of astrocytes (a major type of glial cells in the brain) the post-seizure increase in the expression of gap junctions is well documented [19], suggesting that the same down-regulatory effect as we described here may exist for glia. Gap junctions between astrocytes are presumably needed for efficient transfer of information by inter-cellular calcium waves [34]; however, an excessive expression of gap junctions in astrocyte networks was implicated in seizure initiation [35], [36]. Thus, there is a need for relatively rapid and flexible modulation of the effective gap junction connectivity that would allow efficient propagation of information at the same time preventing pathological dynamics. One solution was recently proposed by assuming that astrocytic gap junctions have nonlinear transfer properties [37]. More studies are needed to find out whether or not the same mechanism applies to neurons and what are its implications regarding collective dynamics in neuronal networks. Meanwhile, the results of the present study call to revise the current dogma regarding the purely pathological role of neuronal gap junctions in epileptic activity.

## Materials and Methods

### Model Network

We described neuronal dynamics using well-studied Morris-Lecar model [23] that was slightly modified by Prescott et al. [24] to account for correct biological mechanisms of action potential generation. This simplified model provides an optimal balance between realistic electrical properties of neuron and computational performance that allows simulations of large-scale 2D networks. Equations that describe the dynamics of model neuron indexed by a pair 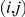 are
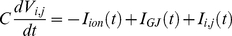
(1)

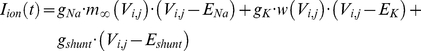
(2)




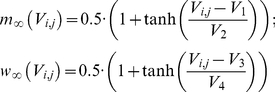
(3)

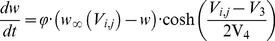
(4)


and are explained in details elsewhere [24]. The following parameter values were used in the model: 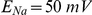, 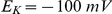, 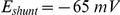, 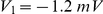, 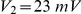,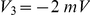, 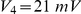, 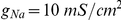, 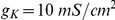, 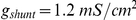 , 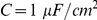, 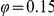.

We consider 2D networks of 50×50 model neurons, with periodic boundary conditions. The term 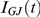 in Equation 1 contains information about the coupling of the neuron 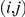 to other neurons
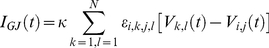
(5)


where 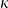 is the strength of the gap junction coupling, and the structure 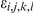 defines the connectivity of the network. Taking as an example the regular 2 dimensional lattice (with Z = 4 model neurons connecting to each model neuron), one has 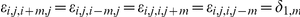 (where 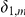 is the Kronecker delta). In this work, we usually considered regular networks with Z = 4,8,12,20,24 peer connections per model neuron (Figure 1A for Z = 4). In some simulations, the effect of adaptive change in gap junction connectivity was tested by creating, in a predefined region, additional connections that targeted neurons beyond the baseline connectivity footprint.

The strength of inter-neuronal gap junction coupling (surface density of gap junction conductance) was set to 
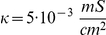
. The exact value of gap junction conductance between a pair of biological neurons is hard to estimate. Amitai et al. [38] estimate that in neocortical interneurons, overall gap junction conductance per neuron (sum over all gap junction conductances to a given neuron) could contribute as much as 50 to 70 percent of neuronal membrane conductance. However, interneurons are known to be much more densely interconnected by gap junctions. Since we do not specify the exact nature of the neurons studied here, we made a modest assumption of individual gap junction conductance density of 
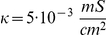
, which is about 0.4 percent of neuronal leak conductance density (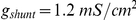).

Activity in biological neuronal network usually arises either due to the synaptic stimulation or as a result of “noise” (this term captures influences such as changes in external milieu, stimulation from glial cells, etc.). Combining realistic topologies of synaptic and gap junction connectivity in the same model framework necessitates accounting for geometrical and morphological aspects (such as the distribution of neurons in space and incorporating dendritic trees), a knowledge that is not fully available at the moment. The goal of the present study was to investigate the contribution of gap junction connectivity to dynamical stability of neuronal ensemble with respect to perturbations in neuronal stimulation. Given this goal, and the complexity associated with the studies of realistic synaptic and gap junction connectivity schemes, we capture the generic effect of synaptic stimulation by introducing, for each model neuron, a non-specific stimulation current
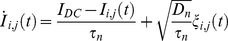
(6)


In Equation 6, 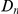 and 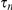 parameterize the intensity of stimulation and the characteristic decay time (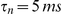), and 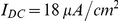 is the mean level of stimulation current. The stochastic variable 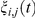 is drawn from temporally and spatially uncorrelated Gaussian distribution
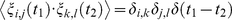
(7)


Collective activity of pyramidal neurons in “healthy” cortex is characterized by relatively low degree of synchrony and individual neuronal firing rates of ∼5 Hz. Therefore, we tuned the parameters of stimulation current to obtain the desired firing rates. With 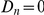, the constant current 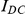 was sub-threshold and no spikes could be generated. Higher firing rate could be obtained by increasing the intensity of the “synaptic” stimulation, 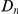 (Figure 1B). Thus, the parameter 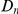 represented deviations, due to the sporadic synchronous events, from asynchronous condition (represented by 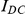). The value of 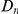 defines the strength of activity perturbation, with higher 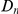 corresponding to stronger perturbation as observed, for example, in epileptic cortices. Conceptually, Equation 6 is equivalent to the Langevin equation for “colored” (correlated) noise [39]; thus, throughout the text we will refer to 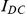 interchangeably as “stimulation current”, “background current” or “background noise”. It is important to note that there is no feedback interaction between the “stimulation current” (representing the effect of chemical synaptic signaling) and the firing activity of neurons in our model network. The potential of gap junctions to contribute to the maintenance of networks' dynamical stability with respect to a perturbation (given by a certain value of 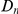) can be deduced by comparing the mean firing rate in different scenarios of gap junction connectivity. Thus, if for a given 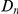, adding gap junctions to a network will reduce the mean firing rate of model neurons, this will be taken as an evidence for network-stabilizing role of gap junctions.

In a separate set of pilot simulations, we explicitly added chemical synaptic connectivity to the model network in order to demonstrate how a localized breach in dynamical stability that arises due to the lesioned gap junction connectivity can ignite the network to exhibit seizure-like activity. To keep the discussion simple, we considered a case when only fast excitatory AMPA synapses were added to the network. Synaptic connectivity was globally random, meaning that each neuron could establish synaptic connection with any other neuron in the network with probability 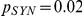. Synaptic current from individual synaptic connection was
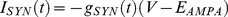
(8)


with 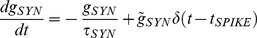
(9)


and 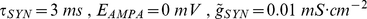.

### Analysis

The firing rate of model neurons was estimated by computing the number of action potentials generated in a predefined time window, and then normalizing by the window duration.

To characterize the extent to which the abundance of gap junctions in a given neuron can affect the propagation of electrical activity, we used the following procedure: First, for a preset time window T, a spike count 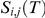 of each model neuron in the network was obtained. Then, for each model neuron, we computed the averaged spike count of its topological neighbors, 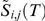. The “spike number disorder” is then defined as
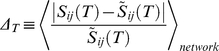
(10)


with the average taken over all model neurons in the network. The measure 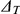 quantifies the normalized absolute deviation of activity from the topological mean. For 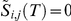 we set 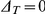.
